# Increased Interpersonal Brain Synchronization in Romantic Couples Is Associated with Higher Honesty: An fNIRS Hyperscanning Study

**DOI:** 10.3390/brainsci13050833

**Published:** 2023-05-21

**Authors:** Chong Shao, Xuecheng Zhang, You Wu, Wenhai Zhang, Binghai Sun

**Affiliations:** 1 School of Psychology, Zhejiang Normal University, Jinhua 321004, Chinaa798774437@outlook.com (X.Z.);; 2 Big Data Center for Educational Neuroscience and Artificial Intelligence, Hengyang Normal University, Hengyang 421001, China; 3 Key Laboratory of Intelligent Education Technology and Application of Zhejiang Province, Zhejiang Normal University, Jinhua 321004, China

**Keywords:** fNIRS hyperscanning, deception, romantic love, gender composition

## Abstract

Previous studies on the brain–brain interaction of deception have shown different patterns of interpersonal brain synchronization (IBS) between different genders. However, the brain–brain mechanisms in the cross-sex composition need to be better understood. Furthermore, there needs to be more discussion about how relationships (e.g., romantic couples vs. strangers) affect the brain–brain mechanism under interactive deception. To elaborate on these issues, we used the functional near-infrared spectroscopy (fNIRS)-based hyperscanning approach to simultaneously measure interpersonal brain synchronization (IBS) in romantic couples (heterosexual) and cross-sex stranger dyads during the sender–receiver game. The behavioral results found that the deception rate of males was lower than that of females, and romantic couples were deceived less than strangers. Significantly increased IBS was observed in the frontopolar cortex (FPC) and right temporoparietal junction (rTPJ) of the romantic couple group. Moreover, the IBS is negatively correlated with the deception rate. No significantly increased IBS was observed in cross-sex stranger dyads. The result corroborated the lower deception of males and romantic couples in cross-sex interactions. Furthermore, IBS in the PFC and rTPJ was the underlying dual-brain neural basis for supporting honesty in romantic couples.

## 1. Introduction

Deception can be defined as a deliberate attempt to mislead others [1]. In the long history of human civilization, interest in this topic transcends most disciplines and cultures [2]. Over the past 100 years, technology has diversified the forms of deception (e.g., e-mails, telephone calls), but the most primitive form, face-to-face, is a more natural context to investigate deception [3]. As interpersonal deception theory (IDT) emphasizes that deception requires human interaction [4], face-to-face deception contains distinct cognitive processes and behaviors that are engaged by both the deceiver and the deceived [5].

Previous studies have revealed gender differences in deceptive behavior, indicating that males cheat more than females, especially with black lies, which benefit the deceiver but cost another person [6,7]. From an evolutionary perspective, this difference can be explained by sexual selection theory, in which males historically faced more substantial incentives and fewer costs for deceptive behavior [8]. As for mixed-gender interactions, evolution theory hypothesized that both sexes have evolved strategies to convey desirable traits to potential partners of the opposite sex [9]. Therefore, since honesty is a desirable trait that both sexes seek in their partner [10,11], less deception will occur in a mixed-gender context. This hypothesis was already supported by a previous study that gender composition systematically affects behavior [12]. However, whether there is a difference in deceptive behavior between the two mixed-gender composition types (male to female vs. female to male) is open to be explored.

On the neural level, two studies have examined interbrain mechanisms underlying face-to-face deception in same-gender dyads [13,14]. Chen et al. [13] found no significant difference in deception rates between male–male and female–female dyads; however, the interbrain mechanisms were different. Specifically, increased interpersonal brain synchronization (IBS) was found in the prefrontal cortex of the female–female dyad and the right temporal–parietal junction (rTPJ) of male–male dyads. In contrast, Zhang et al. [14] found that increased IBS was associated with female–female dyads in the rTPJ. These two studies indicated that deception between the same gender is supported by different brain synchronization mechanisms, and the inconsistencies between the two studies can be explained by differences in the deception paradigm and definition of the IBS. Specifically, Chen et al. adopted a sender–receiver paradigm that comprised both message transfer and verbal statements. This paradigm contrasts the study of Zhang et al. [14], which adopted a gambling card game that did not incorporate these critical elements. Regarding the definition of IBS, Zhang et al. [14] highlighted the comparison between deception and honesty. In contrast, Chen et al. [13] emphasized the dynamic process of deception by comparing the IBS during the deceptive trial to the resting state.

People tend to tell more altruistic but fewer self-centered lies to their lovers than strangers [2,15], who are usually of the opposite sex. However, deception is not always benign in romantic relationships. It can be a double-edged sword. On the one hand, some other-oriented lies can enhance closeness and well-being [16,17]. On the other hand, some malicious lies can damage relationship quality or even cause a breakup [18,19,20,21]. Some researchers have even suggested that the most serious deception occurs between romantic partners. According to attachment theory [22,23], affective bonds in couples can be defined as the emotional tie or bonds of affection that people experience toward their partner, the key feature of which is biobehavioral synchrony. Recently, several hyperscanning studies focused on romantic couples have revealed a unique interbrain mechanism when they engage in interpersonal touch [24,25], social gaze [26], free conversation [27], and cooperation [28,29]. According to these studies, higher IBS is usually associated with better behavioral performance or higher relationship quality, which suggests that IBS may be an effective biological indicator of affective bonds. As with other social interactions, the nature of one’s relationship with another greatly influences the processes of lying and cheating [30]. Previous studies have also shown that deception involving affective stimuli requires more collaboration among brain regions [31]. However, previous studies have not investigated whether affective bonds cause IBS in couples’ interactive deception and its association with behavior. The current study aimed to investigate how mixed-gender composition (male deceiver–female deceived vs. female deceiver–male deceived) and relationship (couple vs. stranger) modulate deceptive behavior and its interbrain mechanisms. In the study, we adapted a sender–receiver game [32], and each pair of participants had to play face-to-face. The informer (i.e., sender, the one who can choose to deceive) has informed the value of the two cards in the game, and the informer can verbally tell the guesser (i.e., receiver, the one who may be deceived) correct or false information about which card is more profitable for the guesser. Based on this message, the guesser makes a decision (which card to choose), determining the informer’s and the guesser’s payoffs. Our paradigm emphasized the ecological validity of experiments, where participants need to engage in rich interactions similar to face-to-face deception in real life.

Based on previous neuroimaging studies, the prefrontal cortex (PFC) is an important area that engages in cognitive processes of deception [33,34,35,36], such as involving working memory, decision making, and executive function systems that inhibit truth-telling, which are necessary for deception. Studies have also shown that increased activation in the PFC is associated with successful deception, suggesting that this region plays a crucial role in regulating deceptive behavior. The prefrontal PFC is also pivotal to the detection of deceptive behavior by processing the cues from the deceiver, such as facial expressions. The right temporoparietal junction (rTPJ) is a brain region strongly associated with mentalizing ability, which is the ability to attribute mental states such as beliefs, desires, and intentions to oneself and others. Numerous neuroimaging studies have reported activity associated with deception in this region [37,38,39]. This is because the deceiver needs to be able to attribute the listener’s mental state to adjust their behavior accordingly. Similarly, for listeners, mentalizing also plays an important role in the process of identifying deceptive behavior. Mentalizing allows the listeners to infer the mental states of the deceiver and make inferences about their intentions and motivations for engaging in deceptive behavior. Due to the fact that our experimental design involves a dynamic interactive process between the informer and the guesser, we have selected these two brain regions as regions of interest. Based on previous studies, our hypotheses were as follows: (i) Males deceive more than females, and romantic couples deceive less than strangers. (ii) Increased IBS would appear in romantic couples, and the deception rate should be negatively associated with increased IBS. This study can provide novel insights into how gender composition affects deception and enhance our understanding of the interbrain mechanisms of romantic couples in social interactions.

## 2. Materials and Methods

### 2.1. Participants

One hundred and sixty-eight college and graduate students participated in this study (84 males/84 females, mean age: 22.0 ± 2.5 years). They were all male–female dyads, including 46 couples and 38 heterosexual stranger dyads. According to the different gender roles in the experiment, the couple dyads were randomly divided into two groups: 23 male informer–female guesser dyads (Couple Male informer–Female guesser dyads, CMF dyads) and 23 female deceiver–male deceived dyads (Couple Female informer–Male guesser dyads, CFM dyads). The romantic relationship duration of the couples lasted for at least three months, but there was no difference between these two couple groups (10.82 ± 2.17 vs. 14.52 ± 2.10 months, *t* (44) = −1.22, *p* = 0.31). Furthermore, couple dyads were asked to complete the Passionate Love Scale (PLS) before the experiment to ensure they were indeed in a romantic relationship. The PLS is designed to measure one’s fascination, desire, and emotional intensity toward another person. Higher scores indicate deeper love [40]. All the participants in the couple dyads had a high level of passionate love (118.35 ± 11.60), and there was no difference between the two groups (*t* = −0.40, *p* = 0.69).

Like couple dyads, 19 male deceiver–female deceived dyads (Stranger Male informer–Female guesser dyads, SMF dyads) and 19 female deceiver–male deceived dyads (Stranger Female informer–Male guesser dyads, SFM dyads) were randomly created in stranger dyads. Gender composition was not a within-subject variable in our experiment, because switching in-game roles would prolong the experimental time and create fatigue and discomfort for the participants. This could have a negative impact on the quality of our data. Therefore, we let participants keep their in-game roles throughout the experiment. There were no age differences between the four groups (*F* (3) = 1.35, *p* = 0.26). All participants were right-handed and had normal or corrected-to-normal vision; none reported a history of psychiatric or neurological disorders. After the experiment, each participant received a basic reward of 25 CNY (approximately USD 3.5) and an additional bonus based on their performance. All the participants gave written informed consent before the experiment. The study was carried out following the guidelines of the Declaration of Helsinki. The Institutional Review Boards of Zhejiang Normal University approved the research protocol.

### 2.2. Task and Procedure

A card game called ‘natural enemy’ was developed for the experiment, inspired by the ‘sender–receiver game’ and a classic Chinese game called ‘tiger–cudgel–rooster’ (similar to the rock–paper–scissors game). This card game has three patterns of cards: cudgel, rooster, and worm. The cudgel card beats the rooster card but loses to the worm card, while the worm card loses to the rooster card.

**Figure 1:**
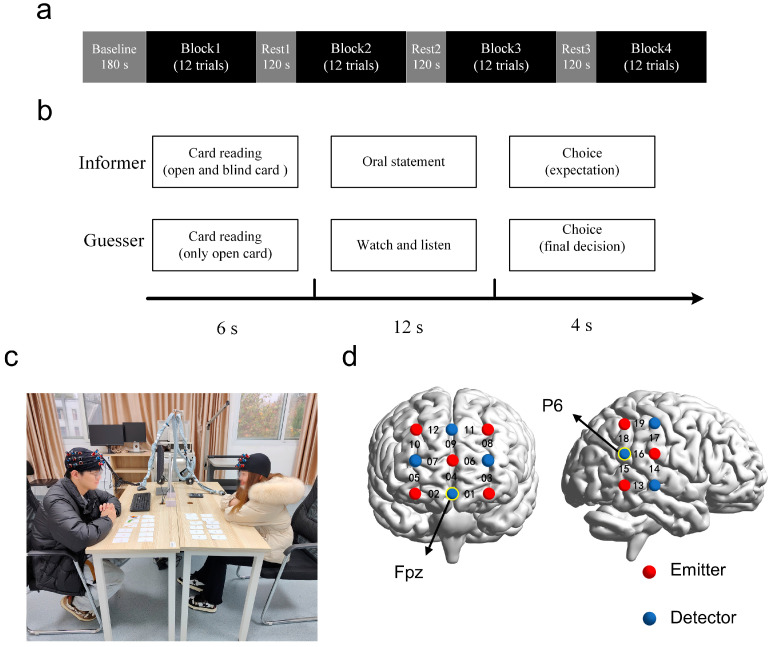
Experimental design. (**a**) Task design. There were four task blocks, each consisting of 12 trials. (**b**) Trial design. Events and time flow in a trial. (**c**) Experimental scene. (**d**) Optode probe set. The optode probes were placed on the prefrontal cortex and the right temporal parietal junction. The Fpz and P6 in the International 10–20 system were used as reference sites.

During the task, an initial 3 min resting-state session served as the brain activity baseline. In this session, participants needed to keep their eyes closed and relax without communication [29]. Then, four blocks of card games were conducted (Figure 1a). Before each block, the experimenter dealt four rows of six cards each on the table between the two participants. All of the cards were facing down. The two rows close to the informer were called blind cards, and the other two rows close to the guesser were called open cards. To avoid confusion, we marked the two types of cards with the numbers 1 to 12 using different colors, meaning that each block contained 12 trials. Participants must complete the whole block according to the number. The cards for each block were prepared in advance and set out on the table in a fixed order that appeared random to the participants.

In this study, two participants in each dyad sat face-to-face on either side of the table and played different roles: informer or guesser. Within each trial (Figure 1b), the guesser was first given 6 s to check the open card and show it to the informer, and simultaneously, the informer needed to check the blind card secretly. Participants were told before the task that the patterns of each pair of open and blind cards were different. After checking both the blind and open cards, the informer had to tell the guesser which of the two cards is higher and give a brief description (e.g., the blind card can beat the open card, it is usually made of wood) within 12 s. The informer could disclose the actual situation or deceive the guesser spontaneously.

After the oral statement, the guesser had the right to choose either of the two cards as the trial’s outcome by pressing keys within 4 s. At the same time, the informer had to answer the question, ‘Which option do you expect the guesser to choose?’ by pressing the keys. The experimental scene is shown in Figure 1c. The guesser would win a token when he/she picked the higher card. Otherwise, the informer would win a token. As an incentive, the participant who won more tokens after all trials received 50 CNY (approximately USD 7), whereas the loser received 25 CNY (approximately USD 3.5). To rule out learning and order effects on the participant’s behavior, the guesser did not know the pattern of the blind cards during the whole experiment, and neither of the participants knew the other’s choice. The time course of the experiment was controlled by voice prompts generated via an E-Prime program.

The informer’s behaviors in the task were classified into three types. (a) Honesty: The informer told the truth and expected the receiver to choose the more profitable option. (b) Deception: Two types of behaviors were defined as deception in this task. Among them, one is that the informer misstated the situation and expected the guesser to pick the lower card; the other is that the informer tells the truth but expects the guesser not to follow it. (c) Undefined: The informer provided false information but expected the guesser to choose the real information. In our study, we focused on analyzing the trials related to deception.

### 2.3. fNIRS Data Acquisition

A multichannel fNIRS system (ETG-4000, Hitachi Medical Corporation, Tokyo, Japan) with a sampling rate of 10 Hz was used to record the brain oxygenation for each dyad. Two 3 × 3 probe patches (five emitters and four detectors, resulting in 12 measurement channels) were placed over the prefrontal regions for two participants in a dyad in accordance with the international 10–20 system. The lowest probe was placed along the Fp1-Fp2 line, and the middle optode was located at the frontal pole midline point (Fpz). Another two 2 × 3 probe patches (three emitters and three detectors, resulting in 7 measurement channels) were placed on the rTPJ for the two participants in a dyad, with the referenced optode placed on P6 in the International 10–20 system [28,41,42]. Therefore, there were 19 measurement channels (CHs), and all emitters and detectors were 3 cm apart (Figure 1d).

We used a three-dimensional electromagnetic tracking device to confirm the spatial position of the emitter and detector (FASTRAK; Polhemus, Colchester, VT, USA). The NIRS-SPM toolbox was used to obtain the Montreal Neurological Institute (MNI) coordinates of the emitters, detectors, and channels. Then, we determined the corresponding brain areas below the channels [43], and the details can be seen in Table S1 in the Supplementary Materials.

### 2.4. Data Analysis

#### 2.4.1. Behavior Performance

The deception rate and the winning rate of the guesser were collected. Two dyads in the CMF group and one in the CFM group were excluded from all further analyses because they did not have any deceptive trials. The remaining participants had at least three deceptive trials (ranging from 3 to 48), and the descriptive result of the deception trials included in the subsequent analysis for each group can be seen in Table S2 in the Supplementary Materials.

The two indexes used SPSS 23.0 (IBM Corp., Armonk, NY, USA) for statistical analysis with an alpha value set to *p* < 0.05. Partial eta squared (*η_p_*^2^) and Cohen’s D were used to evaluate the effect size. Two-way between-subjects ANOVAs were performed with relationship type (couple dyads vs. stranger dyads) and gender composition (male–female vs. female–male) to estimate their effect on task performance.

#### 2.4.2. Interpersonal Brain Synchronization

Based on the modified Beer–Lambert law, the optical data were transformed into changes in the concentrations of oxyhemoglobin (HbO) and deoxyhemoglobin (HbR). Because previous studies showed that HbO signals are more sensitive to changes in cerebral blood flow than HbR [44,45], this study only focused on HbO concentration.

In the first step of preprocessing, the data from the initial 15 s of the rest session were removed to acquire data in the steady-state period. Next, we checked data quality by visual inspection. Channels not showing a clear heart band at approximately 1 Hz in the wavelet transform plot were determined to be bad channels [46], which resulted in 90.32% of the original channels being further analyzed. When more than half of the channels were identified as bad channels, the dyad was excluded from further analysis [47]. In our experiment, no dyad was excluded in this step. Then, each dyad’s data were preprocessed using the NIRS-KIT MATLAB (v2.0) package for detrending and motion correction [48]. Specifically, we first used a polynomial regression model to estimate a linear or nonlinear trend and subtracted it from the raw hemoglobin concentration signal. Then, correlation-based signal improvement (CBSI) was used to reduce motion artifacts [49]. Finally, we used the noise regression functionality to remove global physiological noises such as skull skin blood flow [50].

The wavelet transform coherence toolbox (The MathWorks, Inc., Natick, MA, USA) was used to evaluate the IBS between the informer and the guesser during the whole experiment [51]. Then, the IBS of the oral statement session in deception trials and the baseline (initial resting-state session) were segmented and averaged, and the task-related IBS was defined as the coherence value that the former minus the latter.

**Figure 2:**
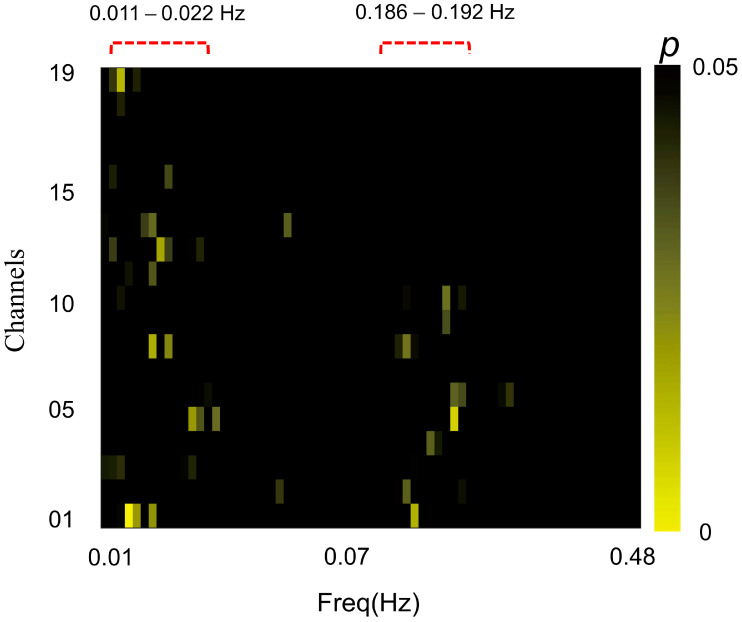
FDR-corrected *p*-maps of couple groups. The task-related IBS of the couple group was significantly higher than that in the resting-state session, ranging between 0.086 and 0.192 Hz (period 5–12 s) and between 0.011 and 0.022 Hz (period 44–94 s). Data values above 0.3 Hz were excluded. The red dashed lines represent the frequency band of interest.

Statistical tests on IBS increases were conducted across the full frequency band to determine the frequency of interest [52,53]. Specifically, we performed a series of one-sample *t*-tests on task-related IBS across the full frequency range to determine the frequency of interest task-related IBS (0.01–1 Hz). The IBS was converted to Fisher *z* score in advance, and the *p* values yielded from the analysis were all with a corrected false discovery rate (FDR). Then, we excluded data values above 0.3 Hz and those below 0.01 Hz to prevent high-frequency noise (e.g., cardiac activity, approximately 0.8–2.5 HZ, and respiration, approximately 0.20–0.30 Hz) and low-frequency fluctuations [54,55]. The above steps were first performed for all subjects, but we did not identify clear significant bands. Therefore, we performed the same analysis separately for the couple and stranger groups. Finally, we found that the task-related IBS was significantly higher than the resting-state session ranging between 0.086 and 0.192 Hz (period 5–12 s) and between 0.011 and 0.022 Hz (period 44–94 s) in the couple group (Figure 2), but there was no clear significant band in the stranger group. Therefore, these two frequency ranges were chosen as our study’s frequency of interest. We further averaged the task-related IBS within the above two frequency ranges, and a one-sample *t*-test was conducted across each channel with FDR correction. The BrainNet Viewer toolbox was used to visualize the *T*-maps on the brain [56]. To further clarify whether the IBS was influenced by the relationship type and gender composition, two-way ANOVAs for IBS at all channels were also performed. Pearson correlation analyses were conducted between behavior indexes (i.e., the informer’s deception rate and the guesser’s winning rate) and significantly increased IBS at channels in each group.

#### 2.4.3. Directional Coupling

We further performed Granger causality analysis (GCA) to estimate the magnitude of the bidirectional information flow between the informer and the follower in deceptive behavior. Since the Granger causality value of neural signals may represent the strength of interpersonal influence during social interaction, it has been widely used in previous fNIRS hyperscanning studies [53,57,58,59]. In our study, GCA was performed for the channels with significantly increased task-related IBS to examine the synchronization direction. Specifically, we extracted the IBS of the oral statement session within the deception trials from the whole time series. Then, we concatenated all the extracted data to create two groups of new time series: informers and guessers. Next, a HERMES MATLAB packet [60] was used to conduct GC estimation in two directions (from the informers to the guessers and from the guesser to the informers). Finally, one-sample *t*-tests were used to examine whether each direction differed from zero, and two-sample *t*-tests were used to compare the differences between the two directions [29].

## 3. Results

### 3.1. Behavioral Performance

**Figure 3:**
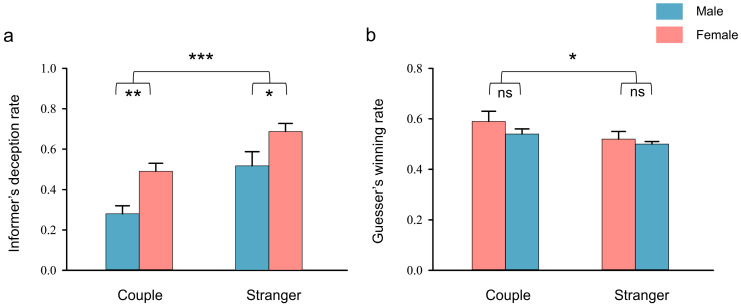
Behavioral results. (**a**) Informer deception rate and (**b**) guesser winning rate in the four groups. Error bars indicate standard errors. * *p* < 0.05, ** *p* < 0.01, *** *p* < 0.001. ns: no significance.

For the informer’s deception rate, the two-way ANOVA showed the significant main effects of relationship type (*F* (1, 77) = 21.49, *p* < 0.001, *η_p_*^2^ = 0.22), with couple dyads showing a lower deception rate than stranger dyads. The significance of gender composition was also observed (*F* (1, 77) = 15.60, *p* < 0.001, *η_p_*^2^ = 0.17), with females deceiving more to males than males to females. There was no interaction between relationship type and gender composition (*F* (1, 77) = 0.09, *p =* 0.76) (see Figure 3a).

For the guesser’s winning rate, the two-way ANOVA showed the significant main effects of relationship type (*F* (1, 77) = 4.07, *p* = 0.047, *η_p_*^2^ = 0.22), with informers in couple dyads showing a higher winning rate than in stranger dyads. There was no main effect of gender composition (*F* (1, 77) = 3.58, *p* = 0.062) or interaction effect between relationship type and gender composition (*F* (1, 77) = 0.001, *p* = 0.98) (see Figure 3b).

### 3.2. Interbrain Synchronization

**Figure 4:**
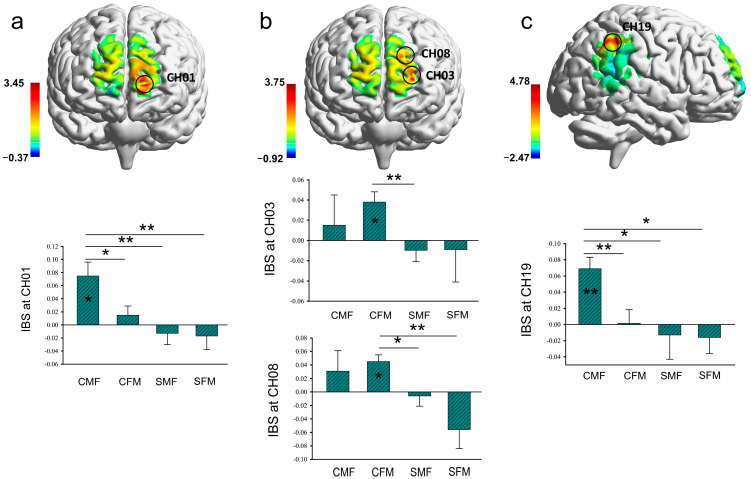
Task-related IBS. (**a**) Upper graph: One-sample *t*-test map of task-related IBS (0.086–0.192 Hz) for the CMF group (false discovery rate (FDR)-corrected). Lower graph: Comparisons of IBS at CH01 (0.086–0.192 Hz) for the CMF group with the other three groups. (**b**) Upper graph: One-sample *t*-test map of task-related IBS (0.086–0.192 Hz) for the CFM group (false discovery rate (FDR)-corrected). Lower two graphs: Comparisons of IBS at CH03 and CH08 (0.086–0.192 Hz) for the CMF group with the other three groups. (**c**) Upper graph: One-sample *t*-test map of task-related IBS (0.086–0.192 Hz) for the CMF group (false discovery rate (FDR)-corrected). Lower graph: Comparisons of IBS at CH19 (0.011–0.022 Hz) for the CMF group with the other three groups. * *p* < 0.05, ** *p* < 0.01.

A series of *t*-tests showed that in both 0.086–0.192 Hz and 0.011–0.022 Hz, task-related IBS was significantly increased only in the couple group (all FDR-corrected). Specifically, at the 0.086–0.192 Hz band, CMF dyads showed significantly increased IBS at CH01 (*t* (20) = 3.45, *p* = 0.048, Cohen’s *d* = 0.75) (see Figure 4a). Moreover, CFM dyads showed significantly increased IBS at CH03 (*t* (21) = 3.56, *p* = 0.017, Cohen’s *d* = 0.76) and CH08 (*t* (21) = 3.75, *p* = 0.017, Cohen’s *d* = 0.80) (see Figure 4b). The above three channels are all located at the Frontal_Sup_L. Furthermore, at the band of 0.011–0.022 Hz, significantly increased IBS was found only in CMF dyads at CH19 (*t* (21) = 4.56, *p* = 0.002, Cohen’s *d* = 1.04) located at the Parietal_Inf_R (see Figure 4c; more details see Table S3 in Supplementary Materials). The difference in the IBS between the significantly increased group and the non-significant group increased group can be seen in Table S4 in the Supplementary Materials.

Next, we sought to determine whether the increased IBS was modulated by relationship type and gender composition. The results of ANOVAs showed a main effect of relationship type in a large number of channels (i.e., CHs 1, 5, 6, 7, 9, 10, 11, 12, 14, 16, 17, 18 at 0.086–0.192 Hz; CHs 2, 5, 6, 7, 9, 12, 14, 15, 17, 18, 19 at 0.011–0.022 Hz, all FDR-corrected) (see Table S5 in Supplementary Materials). The results also revealed that couple dyads showed more increased IBS than stranger dyads in all significant CHs. There was no significant main effect of gender composition and interaction effect in any CHs.

### 3.3. Interbrain Synchronization and Behavior Performance

**Figure 5:**
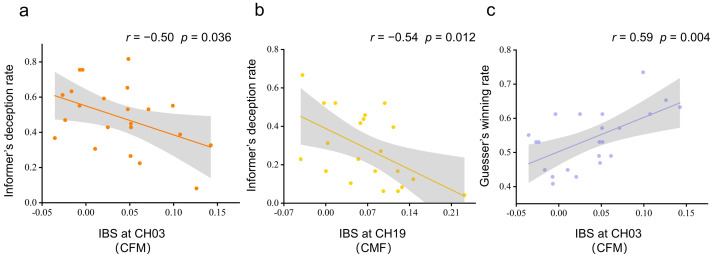
Correlations between behavioral results and IBS. (**a**) Pearson’s correlation between IBS at CH03 and the informer’s deception for the CFM group. (**b**) Pearson’s correlation between IBS at CH19 and the informer’s deception for the CMF group. (**c**) Pearson’s correlation between IBS at CH03 and the guesser’s winning rate for the CFM group. The gray area indicates the 95% confidence interval.

We conducted Pearson correlation analyses of behavioral performance (informer deception rate and guesser winning rate) and significantly increased IBS at channels in the couple group. The deception rate of the informer was negatively correlated with the significantly increased IBS at CH03 in CFM dyads (*r* = −0.50, *p* = 0.036) and CH19 in CMF dyads (*r* = −0.54, *p* = 0.012) (see Figure 5a,b). The positive correlation between the guesser’s winning rate and significantly increased IBS was only in CFM dyads at CH03 (*r* = 0.59, *p* = 0.004) (see Figure 5c).

### 3.4. Directional Coupling

**Figure 6:**
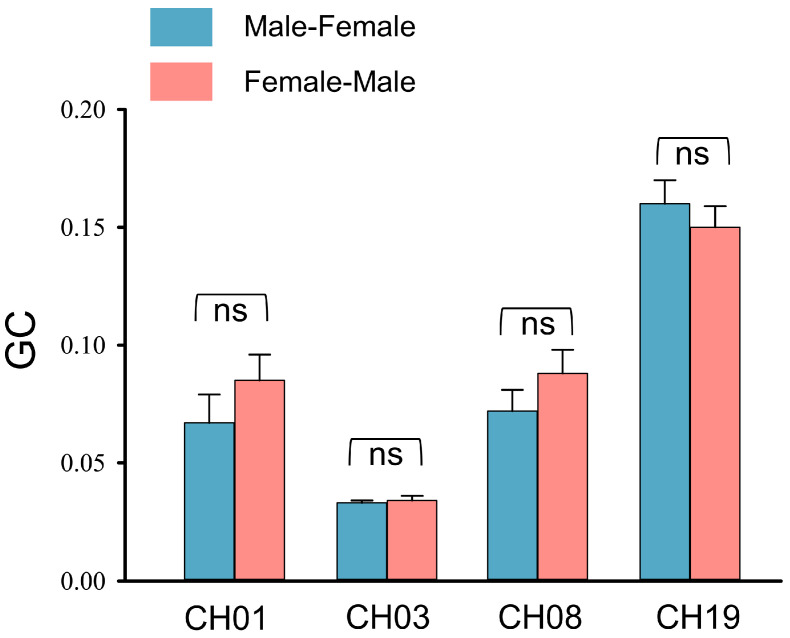
The mean Granger causalities in two directions. Error bars represent standard errors of the mean. ns: no significance.

GCA was conducted to examine the directionality of IBS in significant channels. For all channels, the mean G-causalities of both directions were significantly higher than zero, from the informer to the guesser and from the guesser to the informer (all *p* < 0.05) (see Table S6 in Supplementary Materials). However, two-sample *t*-tests revealed that no significant difference was found between the two directions in all significant channels (CH01, *t* = −1.03, *p* = 0.31; CH03, *t* = −0.46, *p* = 0.65; CH08, *t* = −1.26, *p* = 0.22; CH19, *t* = 1.19, *p* = 0.25) (see Figure 6).

## 4. Discussion

In the present study, we used the fNIRS-based hyperscanning method to investigate how relationship type and gender composition modulate face-to-face deception in a spontaneous context. Here, we found that males deceive females less than females deceive males, and the same is true of romantic couples compared to strangers. The analysis of fNIRS data revealed that romantic couples showed a significantly higher IBS than stranger couples when deception occurred. Furthermore, in romantic couples, the IBS associated with deceptive behavior appeared in different brain regions according to the different gender combinations, but there was no directionality of IBS between the couples. This study deepens our understanding of interactive deception and the underlying dual-brain neural mechanisms of romantic relationships.

One meta-analysis showed that males are significantly more likely to tell lies than females in sender–receiver games [61]. However, we observed lower deception rates among males but not females. The difference between our results and previous studies may be due to the experimental setting. In our study, the target who could be deceived is the opposite sex and sat face to face with the informer. Therefore, gender composition should be taken into consideration. Previous findings have revealed that men cheat on women more often in cross-sex interactions, but cheating is often motivated by mating [62]. They usually try to gain the favor of females by making false promises and bragging about their abilities [8]; that is, the deception of females is a means rather than a purpose, aiming to obtain potential mating opportunities. A large body of research has also indicated that women regard men’s altruism as essential in mate selection, and they rank honesty as more important than men do [63,64]. To promote their self-perceived mate value, men tend to show more honesty, especially in a society with unequal gender roles, such as East Asia [65]. In our study, males’ higher honesty probably implied that they wanted to share resources to show their mating values.

Since honesty and openness are the ideal characteristics of romantic partners, romantic couples tell less self-centered lies than strangers in daily life to maintain a positive relationship [66,67]. In our study, the deception trials indicated a desire for an extra bonus, and against that is the partner’s loss, so it can be seen as self-centered lying. For example, financially related self-centered lies may contribute negatively to romantic relationship satisfaction [68,69]. Our study revealed that romantic couples deceive for extra bonuses much less than strangers, supporting the above view that honesty in romantic couples helps the relationship flourish.

There was no significant increase in task-related IBS in either the SMF or SFM groups, which probably resulted from the gender difference in deception and deception detection at the neural level. Gao et al. [70] reported that in a sender–receiver game, right anodal/left cathodal stimulation of the DLPFC with transcranial direct current stimulation (tDCS) resulted in significantly decreased deception in females but not males, implying that the cognitive process of female deception is more dependent on the PFC. In addition, evidence has shown that females are more skilled in processing facial emotion, which is believed to be critical in deception detection, and the neurobiological basis of this ability is generally located in the PFC [71,72,73]. The hypotheses were supported by a subsequent hyperscanning study that used a similar experimental paradigm to ours, indicating that deception-related IBS was recruited in the PFC only in female dyads [13]. Our work is consistent with and expands on these findings to suggest that deception and its detection have gender differences at the neural level and lead to brain decoupling in interaction deception with the opposite sex.

In our study, we found a significantly increased IBS in romantic couples. Specifically, the IBS in the PFC appeared in both CMF and CFM dyads and was negatively associated with the deception rate in CFM dyads. Studies on the single brain of romantic love have underlined the role of the PFC in avoiding cheating. For example, Ueda et al. [74] reported that individuals in long-term romantic relationships better regulate their interest in dating strangers of the opposite sex with increased activation of the PFC. They proposed that the result reveals an executive control system of the PFC, and the region’s activation positively contributes to romantic maintenance. This is supported by the study of Dogan et al. [75] on the role of PFC, who found that individuals with high honesty have stronger activation of the PFC region when against high economic benefits. Considering the relationship between honesty and trust, a previous study has shown that the brain activation recruited by honesty-based trustworthiness was also shown in the PFC. Moreover, brain signals in this region can predict individual trust in social interaction with their partner [76]. Similarly, in our study, couples were more likely to restrain their desire for monetary rewards and keep honesty, which requires the involvement of executive control. The present findings extend previous findings on the critical role of the PFC in maintaining honesty and trust in dual-brain interactions.

The significantly increased IBS was in the rTPJ in CMF dyads and negatively associated with the deception rate. This result reflected the role of the theory of mind (ToM) in the interaction of couples. ToM is defined as the attribution of others’ mental states, such as desires, intentions, and beliefs [77]. Theory of mind is a prerequisite for forming affective bonds, as it enables individuals to recognize and respond to others’ emotions, needs, and intentions [78], and many findings have highlighted the role of the TPJ in ToM [79,80,81,82]. Previous studies have also shown that focusing on a romantic partner’s mental state recruited more activities in regions that are involved in ToM compared to others, and the network supports the relationship in a positive way [83,84]. In our study, the significantly increased IBS only appeared in the CMF group but not the CFM group, and it is noteworthy that the IBS was detected in the frequency band of 0.011–0.022 Hz, which corresponds to a low-frequency wavelet that captures the slow changing portion of a signal. It suggests the IBS was not specific to the 12 s oral statement stage, but probably the whole task period. A possible explanation for this result is that, compared to the female informers, males had to be more sensitive to females’ emotional states to regulate their behavior in couple dyads. This cautiousness also led to fewer deception trials. In addition, the highest winning rate of female guessers in the couple group indicated that they were more successful in inferring their partner’s intentions. Both sides involved more processes of ToM.

Affective bonds of romantic couples are based on mutual trust and disclosure, which reduce the need and motivation for deception. Couples with strong affective bonds tend to share more information with each other and expect their partners to do the same [85]. Deception can damage the trust and disclosure that sustain affective bonds, thus harming the relationship. Recently, studies have suggested that IBS, as a dual-brain neural mechanism, plays an important role in the generation and maintenance of romantic relationships. Specifically, in a speed-dating task, IBS predicted the outcome of mate choice [86], and for couples in long-term marriages, IBS strongly predicted marital satisfaction when they watched marital movies [87]. We hypothesize that IBS in those studies was the biological basis for affective bonds. The effect was mediated by the more positive ongoing coordination interaction based on honesty and openness between couples.

Consistent with the study of Chen et al. [13], our GCA result found no differences in IBS direction between the informer and guesser in the couple group. The result may be due to the specificity of the face-to-face deception task, which differed from other social interactions, such as instructor–learner [52] or gesturer–guesser interactions [88], indicating that there were no roles of ‘leader’ and ‘follower’ in the interaction, and several previous hyperscanning studies have reached the same conclusion [28,89,90]. This balance can be seen as the synchroneity and equal effort of making inferences about the partner’s mental state.

Several limitations need to be addressed in the present study. First, romantic couples and strangers were included in this study, but we need to consider other relationships, such as intimate friends, to determine whether the findings are specific to couples or influenced by the factor of familiarity. Second, subcortical brain structures, such as the ACC and amygdala, which are also related to deception and romantic behavior, cannot be measured by fNIRS. fMRI- or MEG-based techniques would be feasible to examine those structures further. Third, although the sender–receiver game is an experimental paradigm widely adopted in the study of interaction deception [91,92,93], considering different motivations, emotions, and other factors such as competitiveness and the stage of romantic love, deception between romantic couples is more complicated in real life. Thus, more ecologically valid settings should be developed to investigate this topic. Finally, although widely accepted, it suffers from some limitations due to the baseline setting. A free or specified conversation stage may be a more adequate control than a resting state, since it can exclude the influence of participants’ conversation, eye contact, etc.

## 5. Conclusions

The present study made the first attempt to examine the effect of mixed-gender composition (male–female vs. female–male) and relationship type (romantic couple vs. stranger) on deceptive behavior and related dual-brain connectivity. We found that males had a lower deception rate than females in mixed-gender composition, and compared to strangers, romantic couples had a lower deception rate; significantly increased IBS appeared in the romantic couple group in the PFC and rTPJ and was negatively correlated with the deception rate. These findings contribute to understanding the factors that influence interactive deception and raise an intriguing topic for further research about the role of IBS among romantic couples.

## Data Availability

Data are available for consultation upon request to the corresponding author.

## Supplementary Materials

The following supporting information can be downloaded at: https://www.mdpi.com/article/10.3390/brainsci13050833/s1, Table S1. The coordinates in MNI space and corresponding neuroanatomical labels for channels; Table S2. Descriptive results of the deception trials included in the subsequent analysis for each group; Table S3. The results of one-sample *t*-tests; Table S4. The results of independent-sample *t*-tests on the IBS significantly increased group and non-significant group; Table S5. Two-way ANOVAs on IBS at all the channels in two frequency bands; Table S6. The results of one-sample *t*-tests on Granger causality of two directions in all significant channels.
