# Supplementary material for: Increased Interpersonal Brain Synchronization in Romantic Couples Is Associated with Higher Honesty: An fNIRS Hyperscanning Study
Source: Brain Sci. 2023 May 21;13(5):833. doi: 10.3390/brainsci13050833 (PMC10216105; doi:10.3390/brainsci13050833)
Supplement: Supplementary file 1 [file brainsci-13-00833-s001.zip › brainsci-2334646-supplementary.pdf]

Supplementary materials

Table S1. The coordinates in MNI space and corresponding neuroanatomical labels for channels.

Channel	MNI coordinate			AAL		Brodmann	
	x	y	z	**Area**	**Percentage**	**Area**	**Percentage**
01	-<br>15	72	1	Frontopolar area<br>Orbitofrontal area	0.65<br>0.35	Frontal_Sup_L<br>Frontal_Sup_Medial_L<br>Frontal_Sup_Medial_L<br>Frontal_Mid_Orb_L	0.45<br>0.19<br>0.17<br>0.15
02	17	72	1	Frontopolar area<br>Orbitofrontal area	0.66<br>0.34	Frontal_Sup_R<br>Frontal_Sup_Orb_R<br>Frontal_Sup_Medial_R<br>Frontal_Mid_Orb_R	0.43<br>0.32<br>0.20<br>0.05
03	-<br>26	67	12	Frontopolar area<br>Orbitofrontal area	0.94<br>0.04	Frontal_Sup_L<br>Frontal_Mid_L	0.81<br>0.19
04	5	70	14	Frontopolar area	1.00	Frontal_Sup_Medial_R	0.66
05	29	68	11	Frontopolar area<br>Orbitofrontal area	0.93<br>0.07	Frontal_Sup_R<br>Frontal_Mid_R	0.93<br>0.07
06	-<br>14	66	26	Frontopolar area<br>Dorsolateral prefrontal cortex	0.93<br>0.07	Frontal_Sup_L<br>Frontal_Sup_Medial_L	0.67<br>0.33
07	15	68	26	Frontopolar area<br>Dorsolateral prefrontal cortex	0.98<br>0.02	Frontal_Sup_R<br>Frontal_Sup_Medial_R<br>Frontal_Mid_R	0.51<br>0.42<br>0.07
08	-<br>25	55	35	Dorsolateral prefrontal cortex<br>Dorsolateral prefrontal cortex	0.49<br>0.45<br>0.05	Frontal_Sup_L<br>Frontal_Mid_L	0.60<br>0.40
09	3	59	37	Dorsolateral prefrontal cortex<br>Frontopolar area	0.65<br>0.35	Frontal_Sup_Medial_L<br>Frontal_Sup_Medial_R	0.53<br>0.47

10	28	56	34	Dorsolateral prefrontal cortex<br>Dorsolateral prefrontal cortex	0.51<br>0.41<br>0.08	Frontal_Mid_R<br>Frontal_Sup_R	0.57<br>0.43
11	-11	50	47	Dorsolateral prefrontal cortex	1.00	Frontal_Sup_L<br>Frontal_Sup_Medial_L	0.56<br>0.44
12	15	51	47	Dorsolateral prefrontal cortex	1.00	Frontal_Sup_R<br>Frontal_Sup_Medial_R	0.59<br>0.41
13	69	-50	0	Fusiform gyrus<br>Middle Temporal gyrus<br>Superior Temporal Gyrus	0.42<br>0.38<br>0.13	Temporal_Mid_R<br>Temporal_Inf_R	0.93<br>0.07
14	71	-39	12	Superior Temporal Gyrus	1.00	Temporal_Sup_R<br>Temporal_Mid_R	0.64<br>0.36
15	62	-62	14	Fusiform gyrus<br>Angular gyrus<br>Middle Temporal gyrus	0.58<br>0.20<br>0.14	Temporal_Mid_R<br>Temporal_Sup_R<br>Angular_R	0.91<br>0.07<br>0.02
16	66	-50	27	Superior Temporal Gyrus<br>Supramarginal gyrus<br>Retrosubicular area	0.62<br>0.22<br>0.11	Temporal_Sup_R<br>Angular_R<br>SupraMarginal_R	0.35<br>0.34<br>0.29
17	68	-38	40	Supramarginal gyrus part of Wernicke's area<br>Primary Somatosensory Cortex	0.91<br>0.09	SupraMarginal_R<br>Parietal_Inf_R	0.96<br>0.04
18	59	-60	38	Angular gyrus, part of Wernicke's area<br>Supramarginal gyrus part of Wernicke's area	0.83<br>0.17	Angular_R<br>Parietal_Inf_R	0.70<br>0.30
19	61	-47	50	Supramarginal gyrus part of Wernicke's area<br>Angular gyrus, part of Wernicke's area	0.99<br>0.11	Parietal_Inf_R<br>SupraMarginal_R	0.79<br>0.21

Table S2. Descriptive results of the deception trials included in the subsequent analysis for each group.

Group	*M*±*SD*
CMF	13.62±8.99
CFM	23.86±9.03
SMF	25.40±14.06
SFM	34.00±8.67

Table S3-1. The results of one-sample *t*-tests(CMF group-0.086–0.192 Hz).

Channel	t	p	p_{corr}
01	3.450248	0.002530166	0.048073147
02	-0.37482	0.711738357	0.837832781
03	0.508193	0.616877567	0.781378252
04	0.250783	0.804540235	0.837832781
05	1.576458	0.130606826	0.354504243
06	1.265545	0.220221288	0.516819857
07	0.614147	0.546041772	0.74105669
08	1.048371	0.306972326	0.529631004
09	1.198296	0.244809406	0.516819857
10	0.207351	0.837832781	0.837832781
11	0.988946	0.334503792	0.529631004
12	0.761711	0.455121743	0.665177933
13	3.112305	0.00548948	0.052150063
14	1.917025	0.069632872	0.220504096
15	1.065041	0.299547174	0.529631004
16	2.09166	0.049434764	0.187852104
17	0.223773	0.825204068	0.837832781
18	2.890325	0.00904724	0.057299188
19	2.393307	0.026621151	0.126450468

Table S3-2. The results of one-sample *t*-tests(CFM group-0.086–0.192 Hz).

Channel	t	p	p_{corr}
01	1.063668893	0.29955744	0.517417397
02	0.35091488	0.729148105	0.814930235
03	-0.920313981	0.001829876	0.01738382
04	3.564777567	0.128128968	0.427307854
05	1.584041234	0.184173963	0.427307854
06	1.373205737	0.628817508	0.74672079
07	-0.117103401	0.38214091	0.546793365
08	1.315849811	0.001171283	0.01738382
09	0.490569909	0.296070933	0.517417397
10	0.892686415	0.15099388	0.427307854
11	1.320604854	0.169817818	0.427307854
12	3.752862936	0.402900374	0.546793365
13	1.071577885	0.367860468	0.546793365
14	1.490373864	0.907890869	0.907890869
15	0.239354879	0.813151046	0.858326105
16	2.605925673	0.200845396	0.427307854
17	1.42162262	0.813151046	0.858326105
18	0.853707727	0.016500412	0.104502608
19	0.727416694	0.475003121	0.60167062

Table S3-3. The results of one-sample *t*-tests(CMF group-0.011–0.022 Hz).

Channel	t	p	p_{corr}
01	0.131484806	0.8967055	0.8967055
02	-1.806496382	0.085912191	0.408082909
03	-0.381243282	0.205271824	0.464728707
04	-1.806496382	0.435980878	0.665067639
05	0.794940445	0.14131588	0.447500285
06	0.794940445	0.220134651	0.464728707
07	0.178712294	0.334819488	0.57832457
08	-0.180155922	0.13826294	0.447500285
09	-1.265792012	0.596682833	0.809783844
10	0.988285094	0.082865476	0.408082909
11	-1.085881916	0.022826543	0.216852159
12	-1.544006519	0.45504628	0.665067639
13	0.537750732	0.707042624	0.895587324
14	1.825699732	0.85996155	0.8967055
15	-0.218239031	0.858843055	0.8967055
16	1.296827747	0.290446661	0.551848656
17	-2.466041492	0.829454271	0.8967055
18	-0.761840013	0.209453151	0.464728707
19	4.77820159	0.000114646	0.002178274

Table S4. The results of independent-sample *t*-tests on the IBS significantly increased group and non-significant group.

Channel	Significant group	Non-significant group	*t*	*p*
CH01	CMF	CFM	2.33	0.025
		SMF	3.13	0.003
		SFM	3.02	0.004
CH03	CFM	CMF	0.73	0.470
		SMF	3.02	0.004
		SFM	1.49	0.144
CH08	CFM	CMF	0.45	0.656
		SMF	2.62	0.012
		SFM	3.52	0.001
CH19	CMF	CFM	3.06	0.004
		SMF	2.51	0.017
		SFM	2.68	0.011

Note. CMF: couple-male informer-female guesser dyads; CFM: couple-female informer-male guesser dyads; SMF: Stranger-Male informer-Female guesser dyads. SFM: Stranger-Female informer-male guesser dyads.

Table S5. Two-way ANOVAs on IBS at all the channels in two frequency bands.

Freq	Channel	Relationship type			Gender composition		
		F value	*p*	p_{corr}	*F* value	*p*	p_{corr}
0.192-0.186 Hz	01	10.07	<0.001	0.01	3.20	0.08	0.25
	02	0.67	0.42	0.42	1.31	0.26	0.37
	03	2.78	0.10	0.14	7.79	0.01	0.13
	04	2.49	0.12	0.15	0.28	0.60	0.67
	05	7.96	0.01	0.02	3.17	0.08	0.25
	06	4.91	0.03	0.05	2.20	0.14	0.30
	07	8.43	<0.001	0.02	4.77	0.03	0.20
	08	2.81	0.10	0.14	0.11	0.75	0.75
	09	7.17	0.01	0.02	5.81	0.02	0.17
	10	5.36	0.02	0.04	2.24	0.14	0.30
	11	5.87	0.02	0.04	3.26	0.07	0.25
	12	9.48	<0.001	0.01	0.53	0.47	0.55
	13	2.32	0.13	0.16	1.59	0.21	0.35
	14	5.60	0.02	0.04	0.81	0.37	0.48
	15	1.47	0.23	0.24	2.21	0.14	0.30
	16	11.04	<0.001	0.01	1.51	0.22	0.35
	17	11.12	<0.001	0.01	0.78	0.38	0.48
	18	8.96	<0.001	0.01	1.71	0.19	0.35
	19	1.93	0.17	0.19	0.13	0.72	0.75
0.022-0.011 Hz	01	1.26	0.26	0.28	0.19	0.67	0.89
	02	7.50	0.01	0.02	0.41	0.52	0.89
	03	2.68	0.11	0.13	0.63	0.43	0.89
	04	0.60	0.44	0.44	0.21	0.65	0.89
	05	21.89	<0.001	< 0.001	0.10	0.75	0.89
	06	7.01	0.01	0.03	0.54	0.47	0.89
	07	23.14	<0.001	< 0.001	2.20	0.14	0.89
	08	2.45	0.12	0.14	0.26	0.61	0.89
	09	6.02	0.02	0.04	0.04	0.84	0.89
	10	3.38	0.07	0.10	0.62	0.43	0.89
	11	3.89	0.05	0.08	0.06	0.80	0.89
	12	9.42	<0.001	0.01	1.68	0.20	0.89
	13	3.20	0.08	0.10	0.01	0.93	0.93
	14	12.65	<0.001	< 0.001	0.93	0.34	0.89
	15	4.74	0.03	0.06	0.73	0.40	0.89
	16	2.19	0.14	0.16	0.37	0.55	0.89
	17	4.98	0.03	0.06	3.56	0.06	0.89
	18	8.87	<0.001	0.01	0.09	0.76	0.89
	19	4.55	0.04	0.06	2.59	0.11	0.89

Table S6. The results of one-sample *t*-tests on Granger Causality of two directions in all significant channels.

Channel	Informer-Guesser		Guesser-informer	
	t	*p*	*t*	*p*
CH01	5.48	<0.001	7.25	<0.001
CH03	27.03	<0.001	22.38	<0.001
CH08	8.28	<0.001	8.84	<0.001
CH19	20.11	<0.001	17.85	<0.001
